# Supplementary material for: Massive remobilization of permafrost carbon during post-glacial warming
Source: Nat Commun. 2016 Nov 29;7:13653. doi: 10.1038/ncomms13653 (PMC5141343; doi:10.1038/ncomms13653)
Supplement: Supplementary Information — Supplementary Figures 1-11, Supplementary Tables 1-5, Supplementary Methods and Supplementary References. [file ncomms13653-s1.pdf]

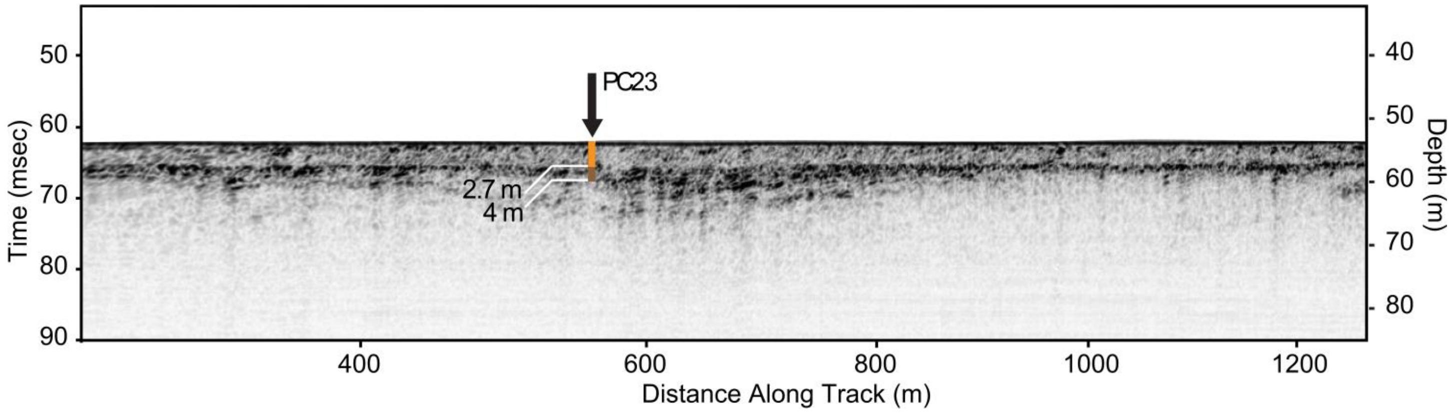

Supplementary Figure 1. Sub-bottom profile of the sampling area. The coring site of PC23 is shown by a black arrow. The seismic profile shows a semi-transparent unit (ca. 2.7 m thick; orange rectangle) above a prominent reflector (brown rectangle) characterized by a greater density consistent with the sediment texture of PC23 (see further details in the text)

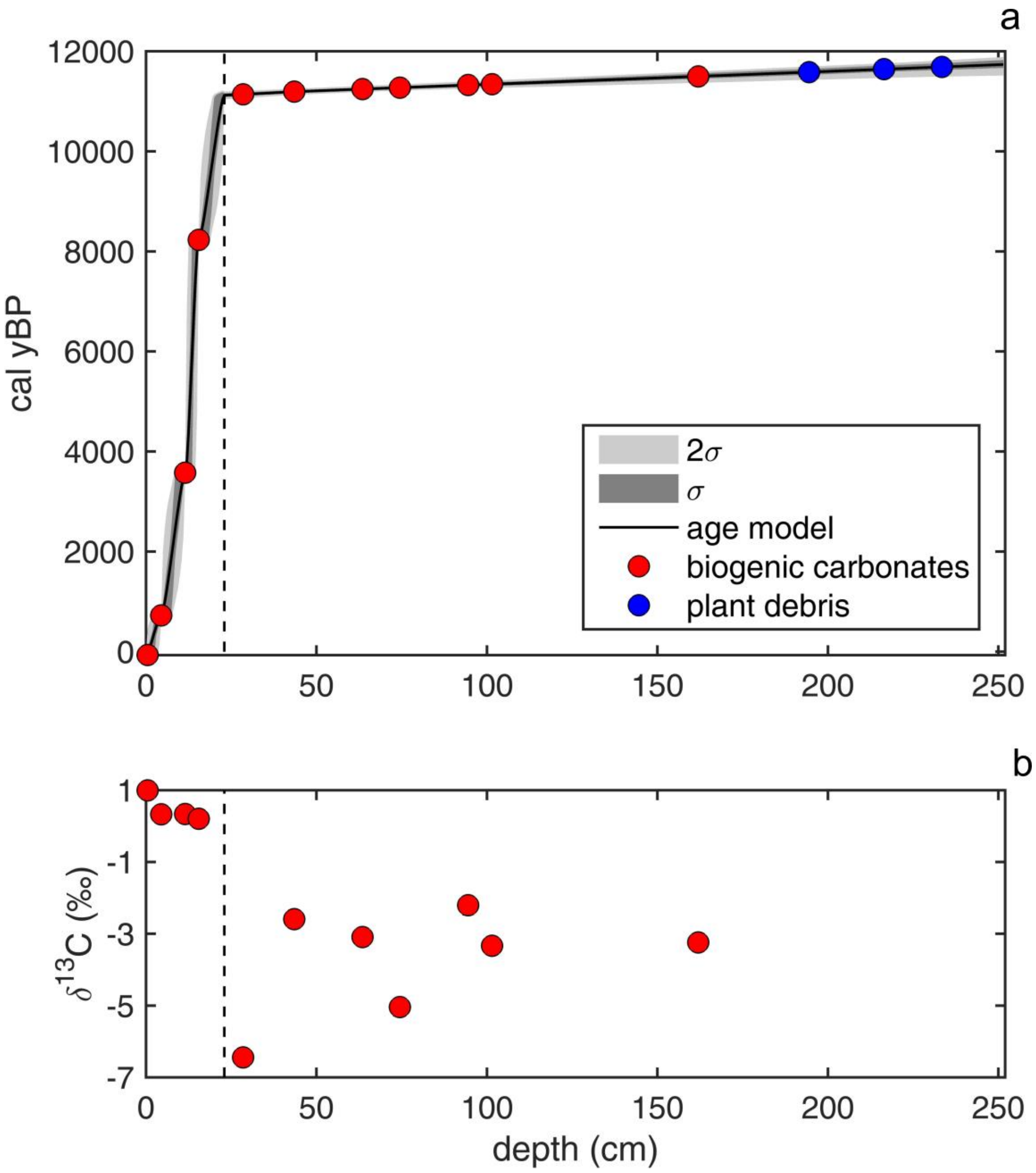

Supplementary Figure 2. Age-depth model and stable carbon isotopic composition of mollusks used in the model. (a) Age model of PC23 based on radiocarbon dating of carbonate fossil remains (shells, red dots) and large vascular plant fragments (blue dots). Light and dark shades of grey show 1σ and 2σ respectively; (b) $\delta^{13}C$ of the carbonates fossil remains used for the dating. The dashed line marks the end of the laminations (see Supplementary Fig. 3) and the change in sediment colour based on the visual description

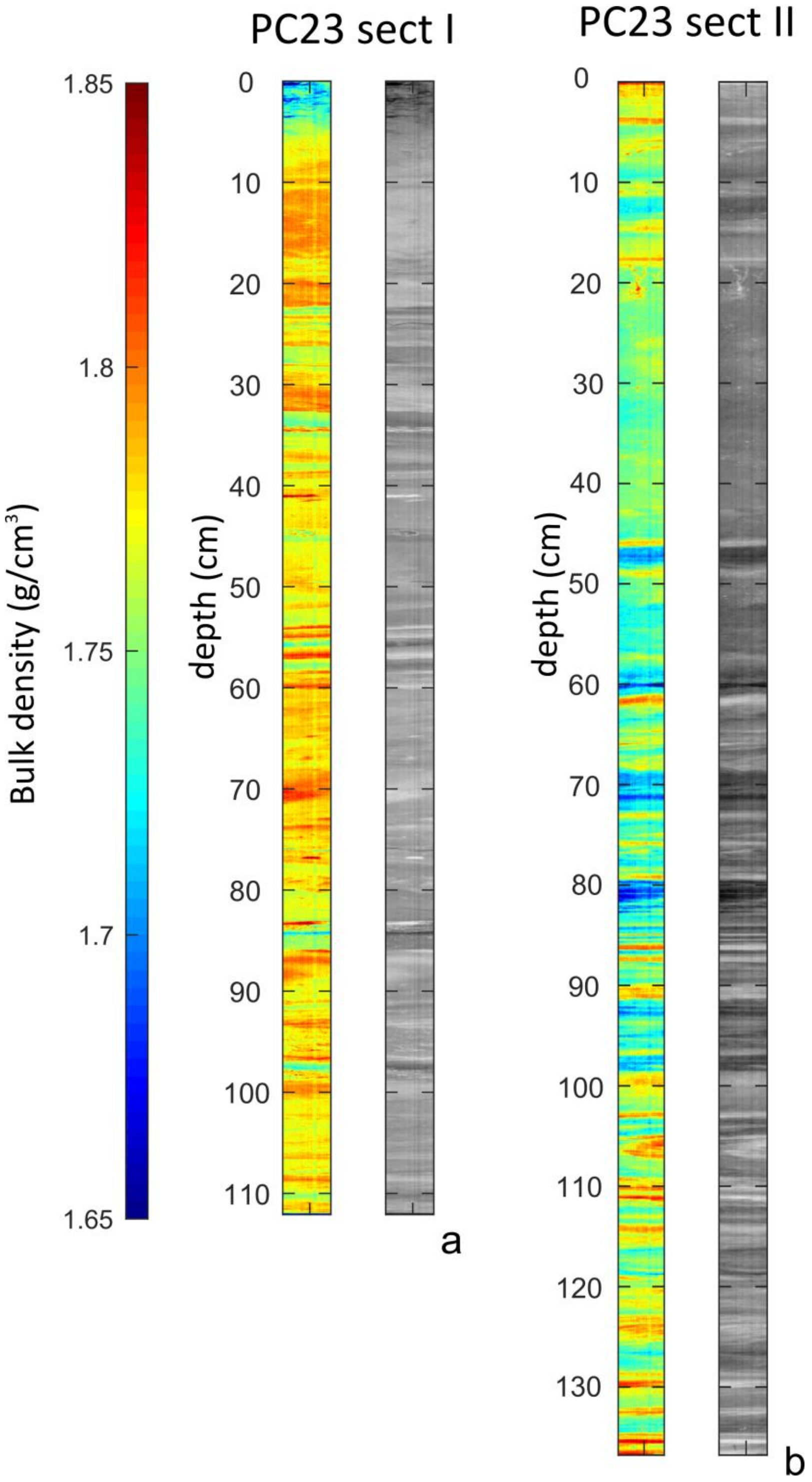

Supplementary Figure 3. X-ray digital radiographs and image analysis of PC23. X-ray digital radiographs showing the sediment bulk density and the internal sediment texture of section I (a) and II (b) of PC23. Colour shading corresponds to the sediment bulk density while grey scale refers to the x-ray digital radiographs

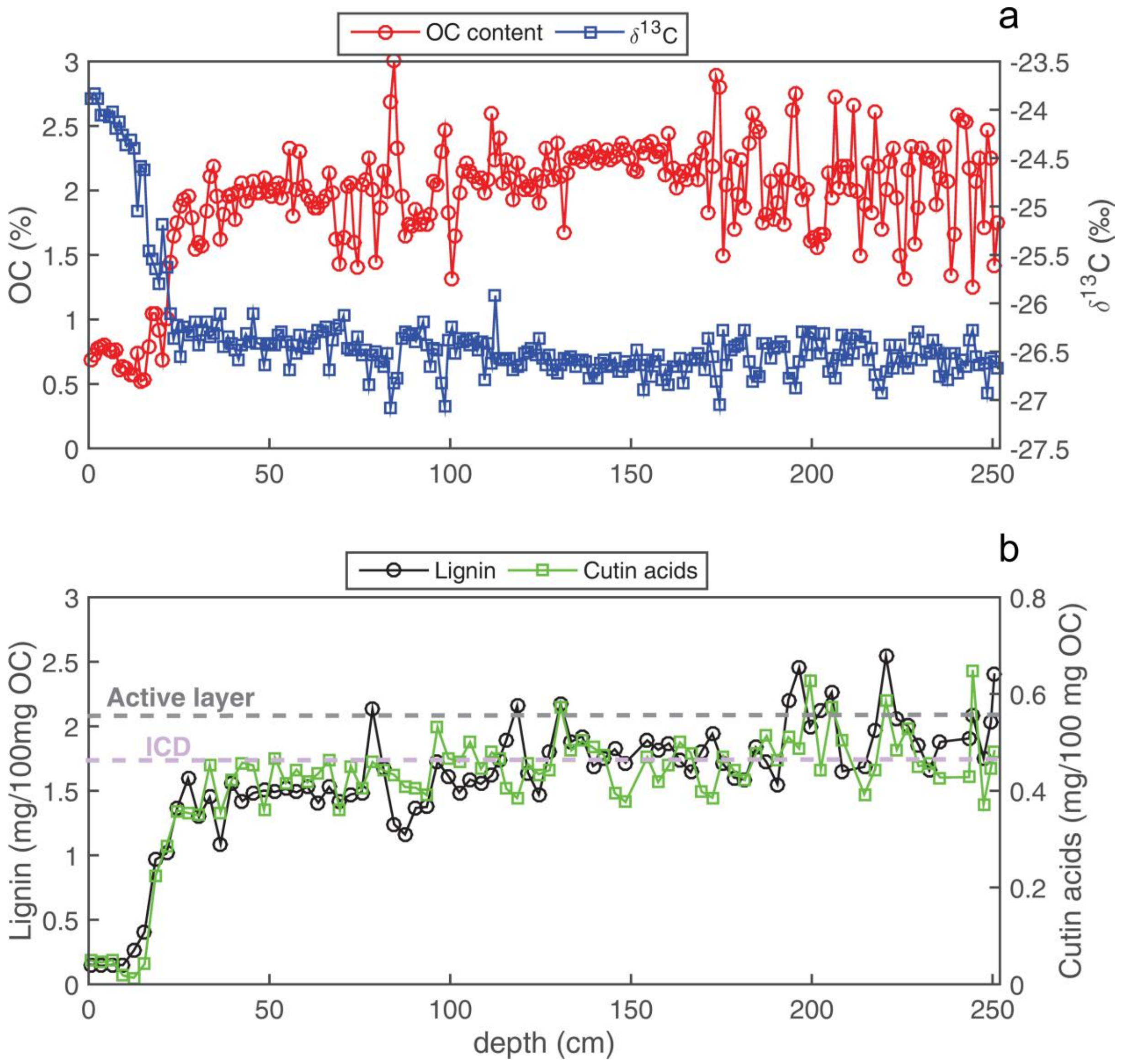

Supplementary Figure 4. Bulk and biomarker composition of PC23. (a) Bulk OC (red line) and $\delta^{13}C$ (blue line), (b) Lignin phenols and cutin-derived products. Average lignin contents in active layer (grey line) and Ice Complex Deposit (ICD, pink line) are shown as dashed lines [1]

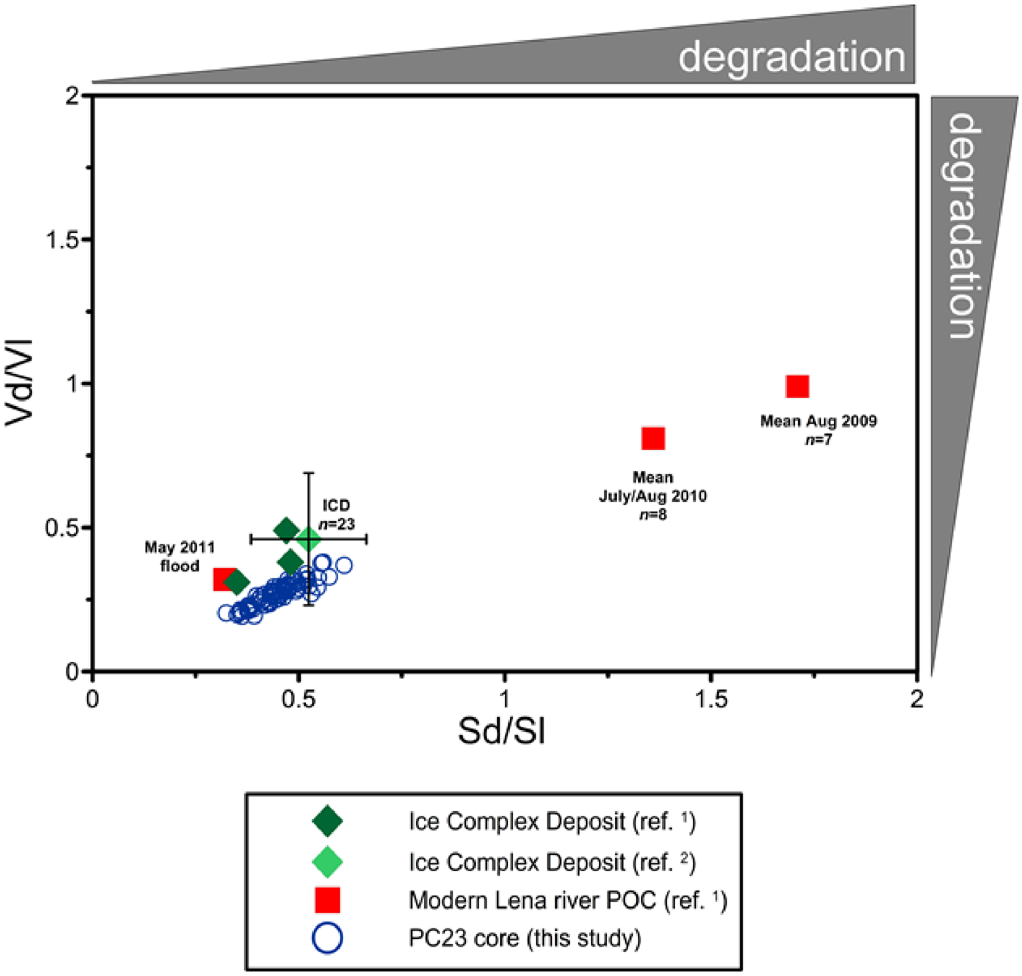

Supplementary Figure 5. Lignin-based degradation proxies. Vd/Vl and Sd/Sl refer to the acid to aldehyde ratios of vanillyl and syringyl phenols, respectively. PC23 (open circles) at the YD-PB transition. As reference, the figure shows the lignin degradation fingerprint of modern particulate material supplied via the Lena in different period (red squares)[2] and Ice Complex Deposit (green diamonds)[1,2]

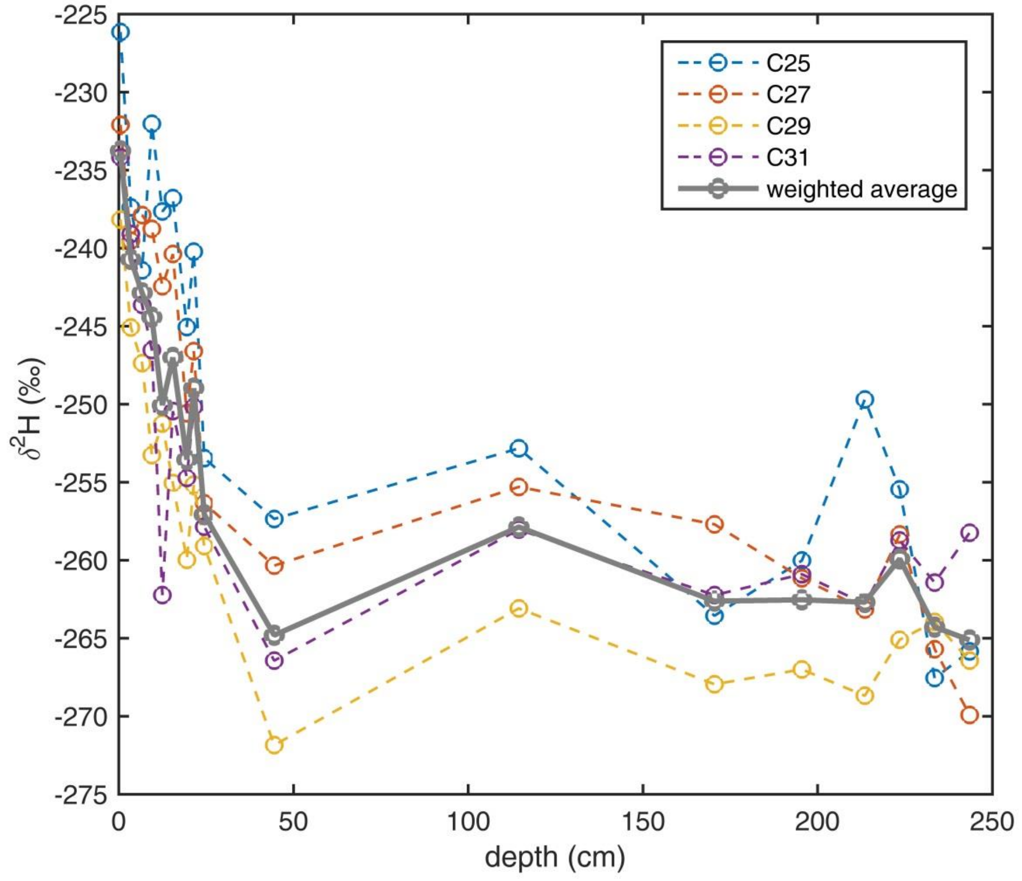

Supplementary Figure 6. Compound specific hydrogen data of PC23. Hydrogen-isotopic abundances (δ^2H) of saturated High Molecular Weight (HMW) odd *n*-alkanes (C25 blue line, C27 red line, C29 yellow line and C31 purple line) in PC23. The grey line shows the weighted average of all HMW *n*-alkanes. For further details about the dataset and the analytical error see Supplementary Table 2

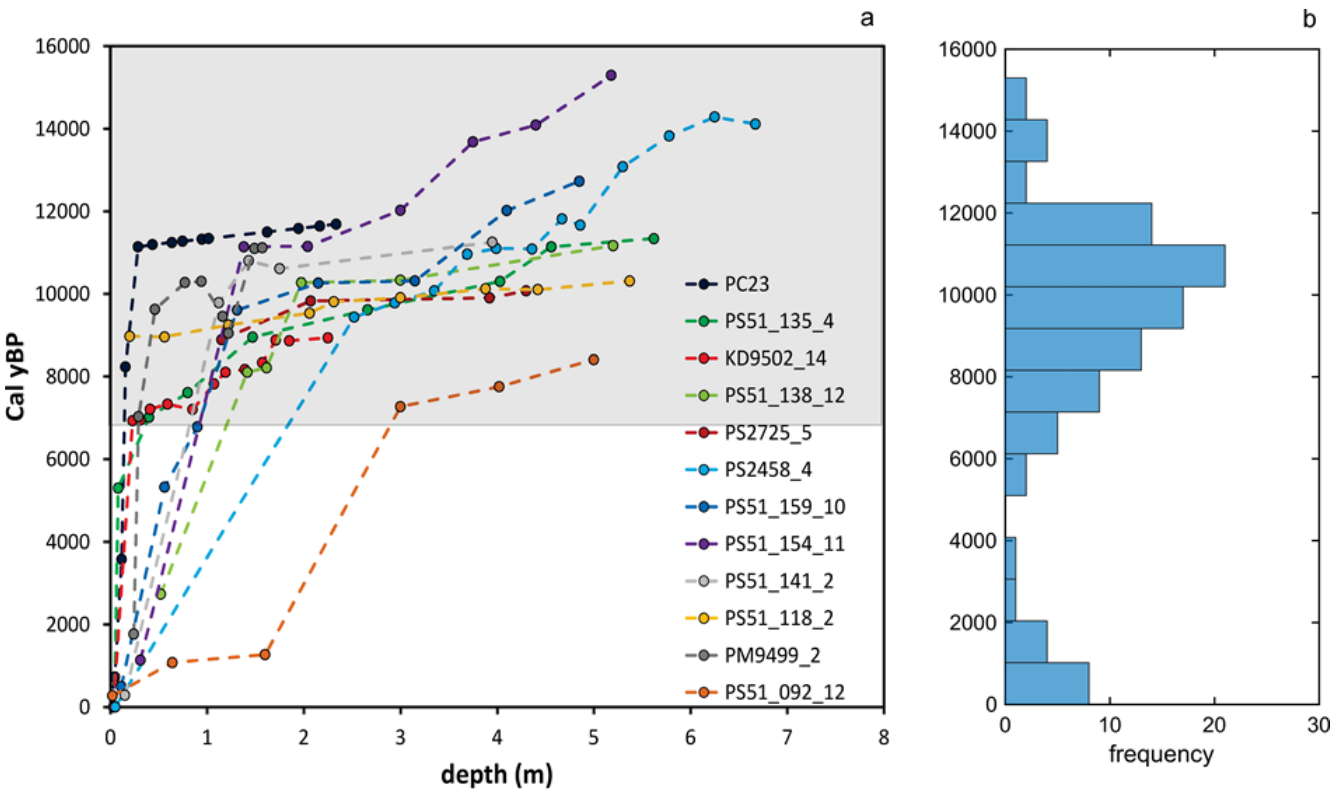

Supplementary Figure 7. Sediment records collected in the Laptev Sea from published studies. (a) Radiocarbon dates of sediment cores collected in the Laptev Sea[3-5]: PC23 (dark blue line), PS51-135-4 (dark green line), KD9502-14 (red line), PS51-138-12 (light green line), PS2725-5 (dark red line), PS2458-4 (light blue line), PS51-159-10 (dark blue line), PS51-154-11 (purple line), PS51-141-2 (light grey line), PS5-118-2 (yellow line), PM9499-2 (dark grey line) and PS51-092-12 (orange line). Location of cores is shown in Fig.1 and Supplementary Fig. 8. Data were used to estimate the accumulation of OC in the Laptev Sea prior to ca. 7,000 yBP (grey box). (b) Histogram showing the distribution of the radiocarbon dates. The histogram shows that the deposit is better resolved between 7,000 and 14,000 cal yBP. See Supplementary Methods (OC burial and flux during last phase of the deglaciation/early Holocene) for further details about the source of the data

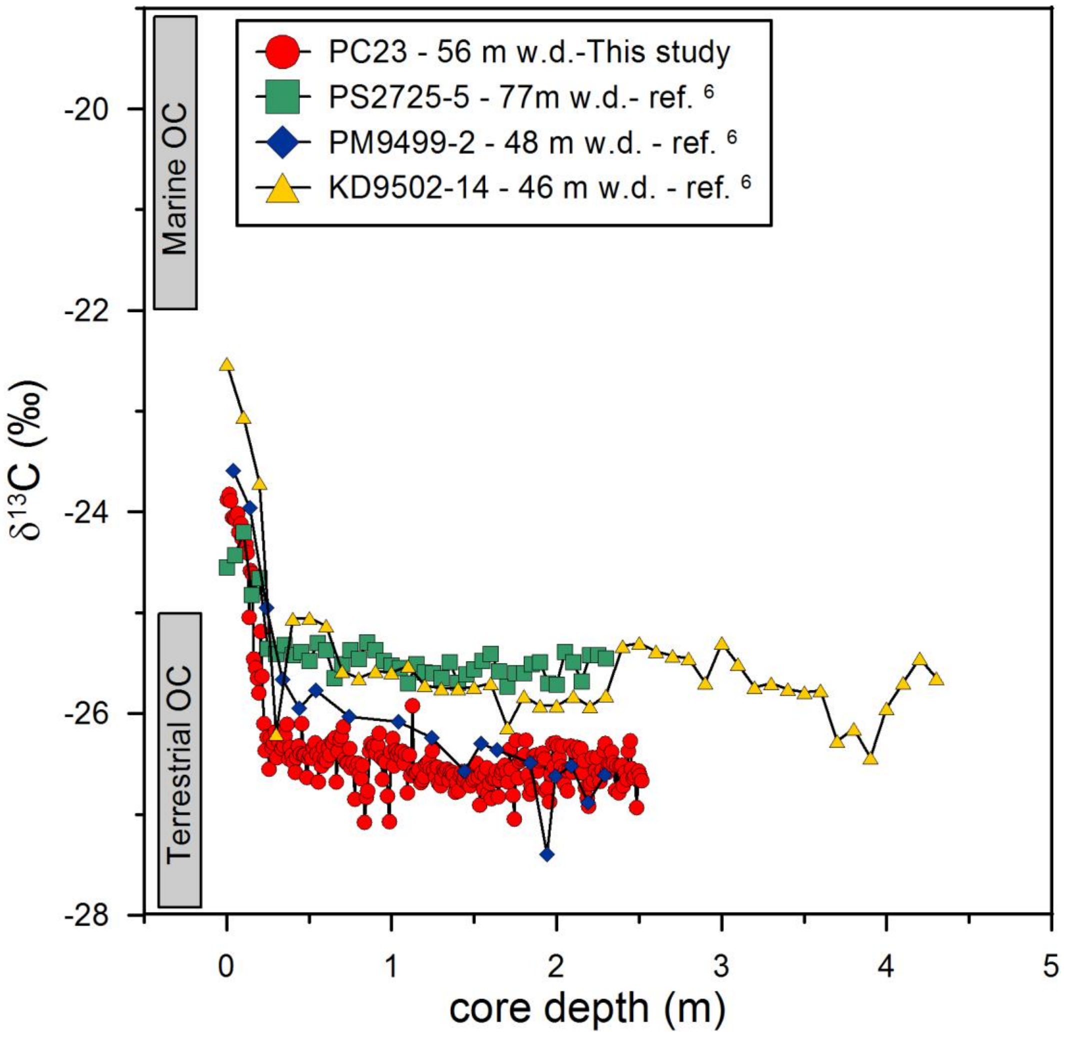

Supplementary Figure 8. Stable carbon isotope composition ($\delta^{13}C$) of organic carbon from sediment cores collected in the Laptev Sea. PC23 (red dots), PS2725-5 (green squares)[6], PM9499-2 (blue diamonds)[6] and KD9502-14 (yellow triangles)[6]. All cores are characterized by a depleted isotopic signature indicating an important influence of terrestrial material. The average composition of marine and terrigenous OC is shown as grey boxes. PC23 is located in the middle of these cores

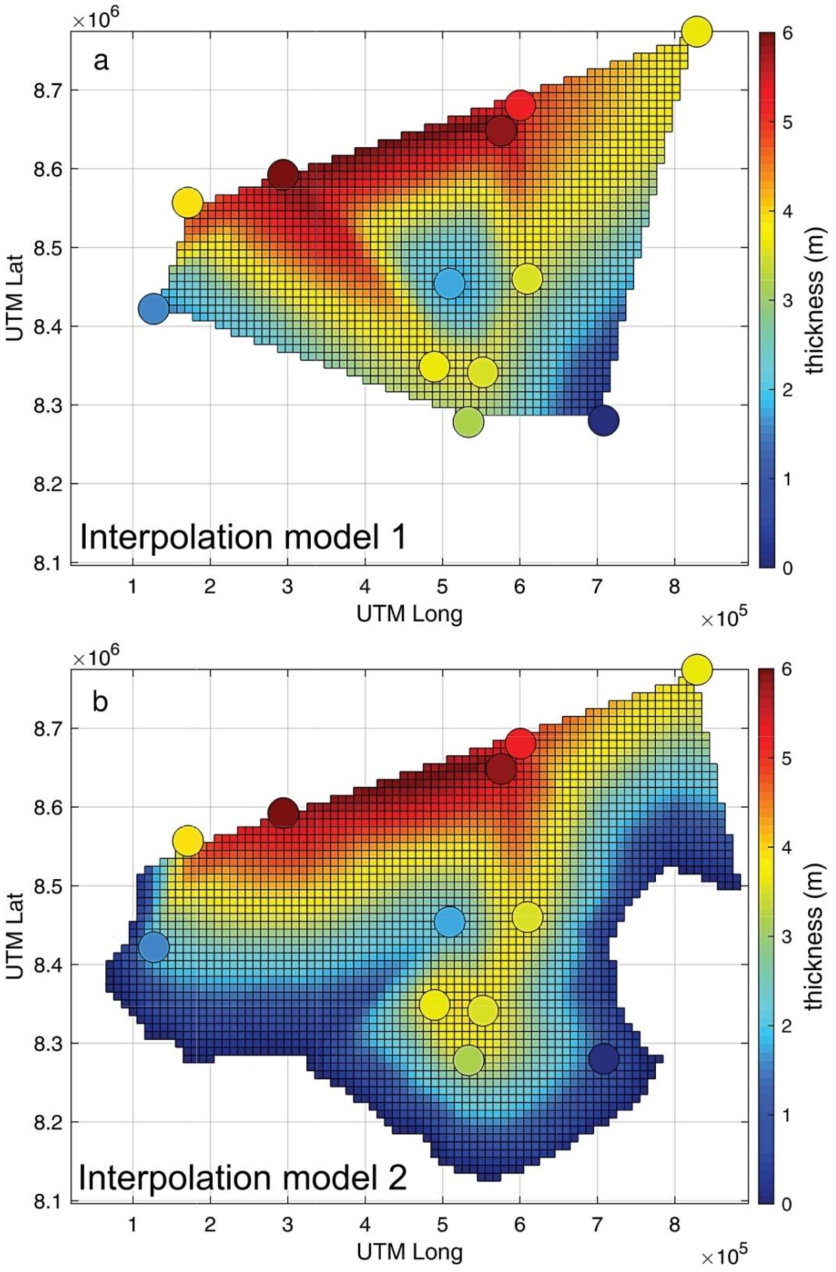

Supplementary Figure 9. Interpolated sediment thickness of transgressive deposits in the Laptev Sea. The thickness refers to the grey box in Supplementary Fig. 6. Coloured circles show the location and thickness of sediment cores presented in the Supplementary Fig. 6. (a) Interpolation model 1; (b) Interpolation model 2. In the Supplementary Methods (see OC burial and flux during last phase of the deglaciation/early Holocene) we provide further details about the interpolation methods

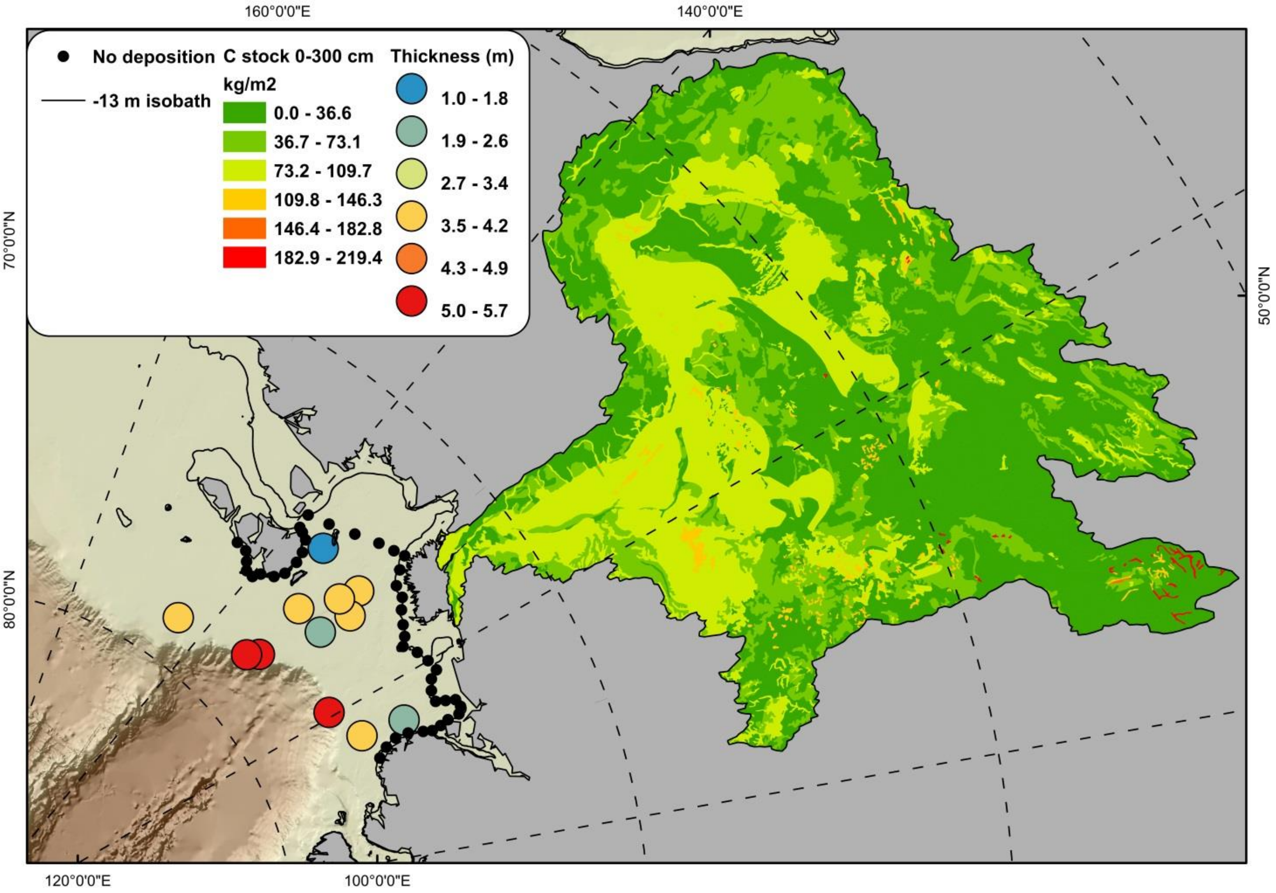

Supplementary Figure 10. OC stock in the Lena River watershed and the thickness of the transgressive deposit. The shading shows the carbon stock in the Lena watershed (Northern Circumpolar Soil Carbon Database)[1] while the open circles displays location and thickness of the transgressive deposits older than 7,000 years as shown in the Supplementary Fig. 6. The black line displays the -13 m isobaths, which was used in the model interpolation 2 (Fig. 7b) as a boundary condition to define the shelf portion without deposition. Black filled circles show the data used in the model for the interpolation where we imposed no accumulation. In the Supplementary Methods (see OC burial and flux during last phase of the deglaciation/early Holocene) we provide further details about interpolation methods and boundary conditions

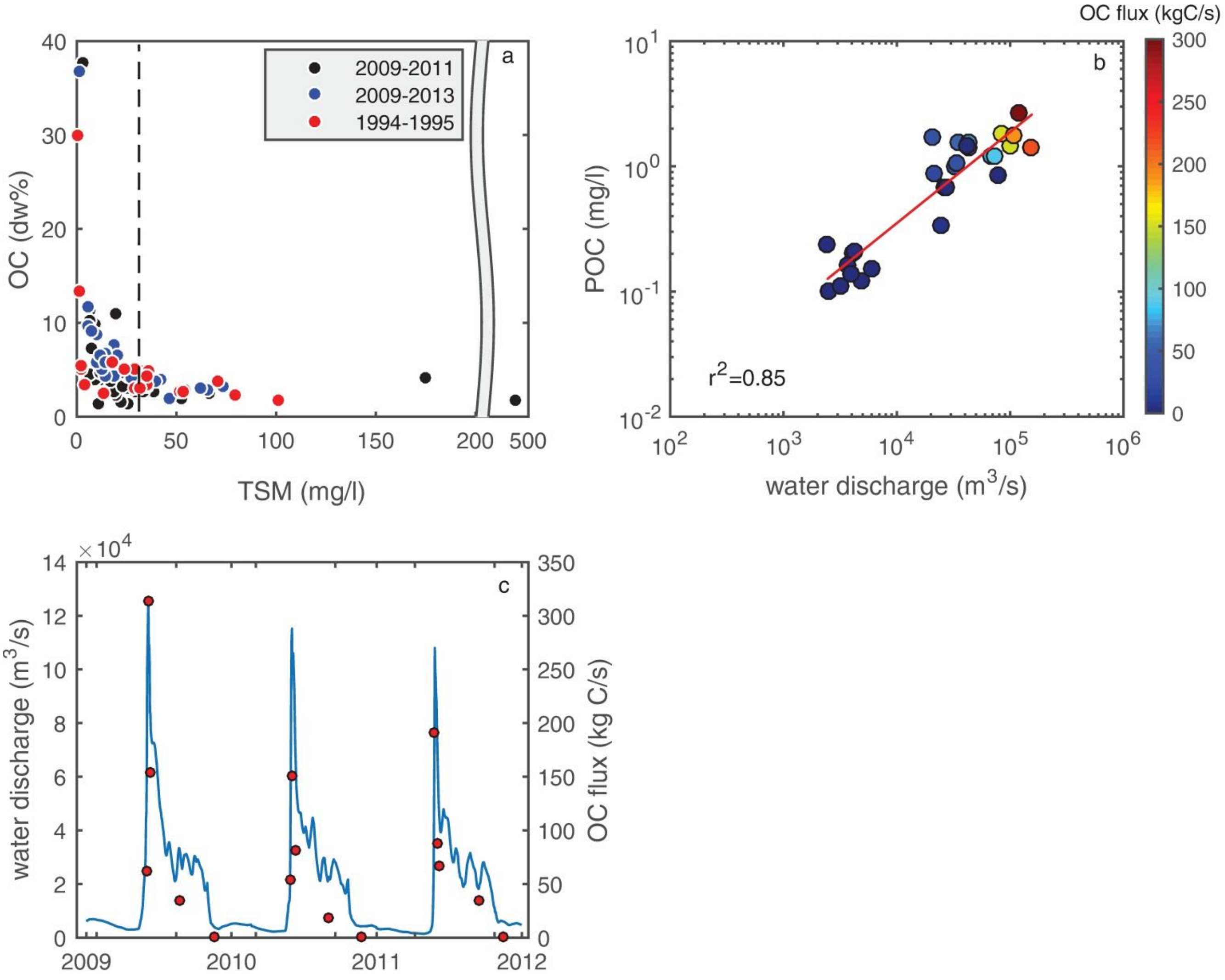

Supplementary Figure 11. Hydrology and particulate OC fluxes of the Lean River. (a) Circles shows TSM and OC of suspended sediments in the Lena river from published (red circles 1994-1995, black circles 2009-2011 and blue circles 2009-2013, *n*=99)[2,7,8].The dashed line marks the lower boundary (30 mg l^{-1}) which was used to calculate the average OC content (% dw) of sediments supplied during high discharge. (b) Rating curve (discharge vs POC) of the Lena River based on surface and depth integrated collection of suspended sediments (*n*=28, Zhigansk gauging station, Arctic-GRO project)[7]. The shading shows the OC flux (c) Relative contribution of high and low flow to the POC transport in the Lena River. Selected three years of measurements at Zhigansk gauging station (Arctic-GRO project)[7]. The blue line shows the discharge while the red dots the OC fluxes

Supplementary Table 1. Radiocarbon data used to develop the age model of PC23

OSAMS ID	ID	depth (cm)	sample type	^{14}C age (yBP)	age err	δ^{13}C (‰)	ΔR	calib ^{14}C age (yBP)	2σ
OS-117287	PC23 I 0-1	0.5	Shell	> modern	20	1.00		-33	-74-471
OS-117402	PC23 I 4-5	4.5	Shell	1240	20	0.33	67	727	634-871
OS-119394	PC23 I 11-12	11.5	Shell	3730	15	0.34	67	3577	3418-3743
OS-117346	PC23 I 15-16	15.5	Shell	7835	30	0.21	67	8326	8068-8367
OS-117403	PC23 I 28-29	28.5	Shell	10440	50	-6.44	400	11140	11064-11209
OS-117347	PC23 I 43-44	43.5	Shell	10730	30	-2.59	400	11193	11131-11254
OS-117488	PC23 I 63-64	63.5	Shell	10710	55	-3.09	400	11246	11187-11310
OS-117348	PC23 I 74-75	74.5	Shell	10740	35	-5.04	400	11275	11215-11340
OS-117641	PC23 I 94-95	94.5	Shell	10840	80	-2.20	400	11326	11255-11398
OS-117628	PC23 I 101-102	101.5	Shell	10710	70	-3.33	400	11343	11267-11420
OS-117404	PC23 II 48-50	162	Shell	10700	50	-3.24	400	11499	11370-11596
OS-116952	PC23 II 81-82	194.5	plant debris	10030	45			11585	11431-11696
OS-116953	PC23 II 103-104	216.5	plant debris	10070	40			11643	11469-11760
OS-116954	PC23 II 120-121	233.5	plant debris	10080	45			11689	11497-11817

Supplementary Table 2. Radiocarbon data of bulk OC used for the source apportionment calculations

NOSAMS ID	ID	depth (cm)	sample type	$\Delta^{14}C$ (‰)	age of deposition (yBP)*	$\Delta^{14}C$ (‰) corrected for the residence time**
OS-118573	PC23 I 0-1	0	bulk OC	-428	-33	-425
OS-118574	PC23 I 4-5	4.5	bulk OC	-510	727	-469
OS-119368	PC23 I 11-12	11.5	bulk OC	-674	3577	-501
OS-118575	PC23 I 15-16	15.5	bulk OC	-757	8326	-340
OS-118576	PC23 I 28-29	28.5	bulk OC	-820	11140	-313
OS-118577	PC23 I 43-44	43.5	bulk OC	-820	11193	-307
OS-118578	PC23 I 63-64	63.5	bulk OC	-818	11246	-295
OS-118721	PC23 I 94-95	94.5	bulk OC	-817	11326	-285
OS-118722	PC23 I 111-112	111.5	bulk OC	-812	11343	-264
OS-118723	PC23 II 48-49	161.5	bulk OC	-814	11499	-260
OS-118724	PC23 II 81-82	194.5	bulk OC	-802	11585	-203
OS-118790	PC23 II 103-104	216.5	bulk OC	-804	11643	-206
OS-118792	PC23 II 120-121	233.5	bulk OC	-815	11689	-244

* From the age model

** Correction for the residence time based on the age of deposition. See Supplementary Methods.

Supplementary Table 3. δ^{2}H data (‰). Mean and standard deviation of saturated odd HMW *n*-alkanes

ID	C25	C27	C29	C31
PC23 I 0-1	-226.1 ±5.9	-232.1 ±1.6	-238.2 ±2.2	-234.2 ±3.7
PC23 I 3-4	-237.4 ±3.4	-239.5 ±2.8	-245.1 ±0.3	-239.1 ±4.3
PC23 I 6-7	-241.4 ±3.0	-237.9 ±2.0	-247.4 ±2.2	-243.6 ±3.0
PC23 I 9-10	-232.0 ±5.9	-238.8 ±2.4	-253.3 ±2.8	-246.5 ±0.5
PC23 I 12-13	-237.6 ±4.1	-242.4 ±3.6	-251.2 ±4.7	-262.2 ±5.0
PC23 I 15-16	-236.8 ±0.9	-240.4 ±3.9	-255.0 ±6.0	-250.4 ±3.3
PC-23 I 19-20	-245.0 ±1.5	-250.6 ±1.9	-260.0 ±1.6	-254.7 ±2.1
PC23 I 21-22	-240.2 ±4.2	-246.6 ±3.6	-255.2 ±2.3	-250.2 ±5.5
PC23 I 24-25	-253.5 ±2.5	-256.3 ±4.8	-259.1 ±3.4	-257.8 ±2.6
PC23 I 44-55	-257.3 ±2.5	-260.3 ±2.9	-271.8 ±2.4	-266.4 ±1.4
PC23 II 1-2	-252.8 ±2.4	-255.3 ±2.0	-263.1 ±1.7	-258.0 ±3.6
PC23II 57-58	-263.5 ±1.9	-257.7 ±1.2	-267.9 ±1.9	-262.2 ±3.2
PC23 II 82-83	-260.0 ±4.8	-261.1 ±1.1	-267.0 ±2.3	-260.9 ±3.7
PC23 II 100-101	-249.7 ±2.9	-263.1 ±1.2	-268.7 ±1.5	-262.8 ±2.2
PC23 II 110-111	-255.5 ±1.1	-258.3 ±2.6	-265.1 ±3.7	-258.7 ±1.5
PC23 II 120-121	-267.5 ±3.2	-265.7 ±2.6	-263.9 ±5.3	-261.4 ±3.4
PC23 II 130-131	-265.8 ±3.8	-269.9 ±2.1	-266.4 ±3.1	-258.2 ±3.9

Supplementary Table 4. Data used in the interpolation method 1 and 2 to estimate the carbon buried in the Laptev Sea during transgression

Core ID	water depth (m)	Lat °N	Long °E	thickness (m)	source
PM9462-4	-27	74.503	136.005	1.0	Ref. 2
PS51/092-12	-32	74.593	130.139	3.5	Ref. 2
PS51/141-2	-42	75.227	128.641	3.9	Ref. 2
PS51/138-12	-45	75.153	130.829	3.8	Ref. 4
PM9499-2	-48	75.501	115.545	2.2	Ref. 2
Average KD9502-14&PS51/135-4	-48	76.192	133.117	3.7	Ref. 2
SWERUS-C3 PC23	-56	76.171	129.337	2.4	this study
PS51/159-10	-60	76.767	116.032	4.1	Ref. 2
PS2725-5	-77	78.656	144.135	3.9	Ref. 3
PS51/118-2	-114	77.892	132.237	5.6	Ref. 2
PS51/154-11	-270	77.276	120.610	5.7	Ref. 2
PS2458-4	-983	78.167	133.398	5.1	Ref. 2

Supplementary Table 5. Fractions of PF-Active layer, PF-Ice Complex Deposit (PF-CD) and Marine OC (Mar OC) in PC23. The table shows mean ± standard deviation based on the Monte Carlo simulation.

Core interval	Depth (cm)	Age (cal yBP)	OC fraction		
			PF-Active-layer	PF-ICD	Mar OC
PC23 I 0-1	0.5	-64	0.10 ±0.09	0.39 ±0.04	0.51 ±0.07
PC23 I 4-5	4.5	727	0.09 ±0.08	0.44 ±0.04	0.47 ±0.07
PC23 I 11-12	11.5	3577	0.13 ±0.1	0.47 ±0.05	0.40 ±0.08
PC23 I 15-16	15.5	8326	0.16 ±0.15	0.30 ±0.06	0.54 ±0.11
PC23 I 28-29	28.5	11140	0.62 ±0.14	0.20 ±0.1	0.18 ±0.1
PC23 I 43-44	43.5	11193	0.59 ±0.15	0.20 ±0.09	0.21 ±0.12
PC23 I 63-64	63.5	11246	0.60 ±0.15	0.18 ±0.09	0.22 ±0.12
PC23 I 94-95	94.5	11326	0.61 ±0.16	0.17 ±0.09	0.22 ±0.13
PC23 I 111-112	111.5	11369	0.65 ±0.15	0.15 ±0.09	0.20 ±0.12
PC23 II 49-50	162.5	11499	0.71 ±0.12	0.15 ±0.09	0.14 ±0.09
PC23 II 81-82	194.5	11585	0.77 ±0.10	0.12 ±0.08	0.11 ±0.08
PC23 II 103-104	216.5	11643	0.76 ±0.11	0.11 ±0.08	0.13 ±0.09
PC23 II 120-121	233.5	11689	0.72 ±0.12	0.14 ±0.09	0.14 ±0.09

SUPPLEMENTARY METHODS

Sampling and sediment core handling

The piston core 23 (PC23) was collected in the mid/outer-shelf of the Laptev Sea (Lat 76° 10.26' N, Long 129° 20.22'E, water depth 56 m; recovery 4.02 meters) in July 2014 during the SWERUS-C3 expedition (*I/B Oden*) (Fig. 1). Sub-bottom profiles acquired in the sampling area reveal a ca. 2.7 m thick acoustically semi-transparent unit above a prominent reflector (Supplementary Fig. 1). Below this reflector the chirp sonar signal does not penetrate much further. Below the semi-transparent unit there is a hint of reflectors slightly unconformable which corresponds to sandy material in the sediment core. This basal sandy deposit was interpreted as reworked transgressional material following the flooding of the shelf. The current study focuses on the upper region of the sediment core which corresponds to the acoustically semi-transparent unit in Supplementary Fig. 1.

PC23 was split on board and half of the core was immediately subsampled at 1-cm intervals for biogeochemical analyses. Sediment subsamples were frozen (-20°C) while the remaining unsampled material was kept at 4°C throughout the expedition. In the lab, prior to biogeochemical analyses, frozen samples were freeze-dried and sieved (500 µm) to isolate biogenic carbonates and plant debris. A few grams of freeze-dried sediments were ground for organic geochemical analyses. Bulk density (ρ_{bulk}) and dry bulk density (ρ_{dry}) were calculated using Eq.1 and Eq.2

$$\rho_{bulk} = W_{wet} \times (V_{sed}+V_{H2O})^{-1} \qquad \text{(Eq.1)}$$

$$\rho_{dry} = W_{dry} \times (V_{sed}+V_{H2O})^{-1} \qquad \text{(Eq.2)}$$

where W_{wet} is the weight of the sample before freeze-drying, W_{dry} is the weight of the dry sample, while V_{sed} and V_{H2O} are the volumes of the dry sediment and the water, respectively. V_{sed} and V_{H2O} were calculated by weighing the samples before and after freeze-drying assuming a dry sediment density of 2.65 g cm^{-3}.

Source apportionment calculations

Markov chain Monte Carlo (MCMC)

$\delta^{13}C$, $\Delta^{14}C$ and OC-normalized lignin content were used to differentiate between three major sources of OC: Permafrost active-layer (PF-AL), Permafrost Ice Complex Deposit (PF-ICD) and marine OC (MarOC) (Fig.3). Under the assumption of source marker mass-balance this system is over-determined, since in principle N+1 sources can be differentiated with N markers:

$$\begin{pmatrix} \delta^{13}C_{sample} \\ \Delta^{14}C_{sample} \\ lignin_{Sample} \\ 1 \end{pmatrix} = \begin{pmatrix} \delta^{13}C_{PF-AL} & \delta^{13}C_{PF-ICD} & \delta^{13}C_{MarOC} \\ \Delta^{14}C_{PF-AL} & \Delta^{14}C_{PF-ICD} & \Delta^{14}C_{MarOC} \\ lignin_{PF-AL} & lignin_{PF-ICD} & lignin_{MarOC} \\ 1 & 1 & 1 \end{pmatrix} \cdot \begin{pmatrix} f_{PF-AL} \\ f_{PF-ICD} \\ f_{MarOC} \end{pmatrix}$$

(Eq.3)

where *f* is the fractional contribution from each source, *sample* refers to the marker values in the sediment core, the 4x3 matrix contain the endmember values, in which the last row assures mass-balance. This type of over-determined system is straight-forward to implement into an existing 3 marker/4 source Bayesian MCMC framework using Eq.3[9,10]. A key advantage of this approach is that the variability of the marker values for each source (endmembers), along with the uncertainties of the estimated relative source contributions, are accounted for.

In these calculations, each endmember was represented by a normal distribution parametrized by literature values for the mean and standard deviation. Computations were run in Matlab using 200 000 iterations, a burn-in (initial equilibration) of 10 000 and a data thinning (to remove step-by-step correlations) of 10. Thus, in total 19 000 numbers of the fractional contributions from each of the three sources were computed. From these, the mean and standard deviation of the relative contributions from each source were estimated. Prior to the calculations, $\Delta^{14}C$ data were corrected to account for the residence time after the deposition using the Eq.4

$$\Delta\,^{14}\mathrm{C} = [\mathrm{Fm} \times e^{\lambda\,(1950-\mathrm{Yd})} - 1] \times 1000 \qquad \text{(Eq.4)}$$

where *Fm* is the Fraction of Modern carbon, λ is 1/(true mean-life) of radiocarbon and *Yd* is the year of deposition (based on the age model). Results are shown in Supplementary Table 5.

Endmember definition

The average and standard deviations of each endmember used for the source apportionment calculations (Fig. 3) were defined by compiling data from the literature.

The ICD endmember, as recently reported[11], consists of 300 $\Delta^{14}C$ values (-940±84‰). For our historical reconstruction, we removed all the dates younger than 11,500 years (the dataset apparently contained some sub-surficial soil intervals as well) to generate a new dataset of 264 $\Delta^{14}C$ values (-966±45‰). This radiocarbon value was then corrected to account for the age of deposition which resulted in different (i.e. younger) ^{14}C value according to the position along the sediment core. Correction was performed using Eq. 4. Finally, the other variables of the ICD consist of 12 $\delta^{13}C$ values (-26.30±0.67‰)[12] and 23 lignin values (1.74±8.3 mg gOC^{-1})[1]; the $\delta^{13}C$ data are in turn the average of 12 different ICD outcrop sites (dataset of 374 $\delta^{13}C$ measurements)[12].

The active-layer was defined by compiling data from the uppermost metre of Siberian soils located in the continuous permafrost domain. The active-layer dataset consists of 29 $\Delta^{14}C$ values (-232±147‰)[2,13-16] and 38 $\delta^{13}C$ values (-26.95±1.17‰)[1,2,13,17-19] and 16 lignin values (2.09±0.64 mg gOC^{-1})[1]. For this study, we primarily focused on the sea-ice algae contribution because during the deglaciation the MarOC flux to the seabed was likely driven by ice-edge algae productivity at the piston core site. Thus the MarOC endmember was defined on the basis of the literature which specifically characterized the sea-ice phytoplankton composition.

The MarOC dataset consists of 5 $\Delta^{14}C$ data (-50±12‰)[20] from the East Siberian Sea and 30 $\delta^{13}C$ data (-20.97±2.56‰)[20-26] from different regions of the Arctic Ocean. As lignin is a macromolecule exclusively produced by vascular plants, its value for the MarOC endmember was set to 0±0 mg gOC^{-1} for the source apportionment calculations.

OC burial and flux during last phase of the deglaciation/early Holocene

The thickness of the transgressive deposit in the Laptev Sea was assessed on the basis of published studies (data available in PANGAEA) in addition to PC23. The full dataset consists of 12 radiocarbon dated piston cores (water depth between ca. 30 and 980 m, Supplementary Fig. 7 and Supplementary Table 4)[3-5]. Geographically, PC23 is located in the middle of the records (Fig.1,). The $\delta^{13}C$ data, available for three records (Supplementary Fig. 8), are consistent with PC23 and confirm the deposition of predominantly terrigenous material, regardless of the core location[6].

We used the original chronologies provided in the published studies[3-5]. In general, each sediment record shows the same pattern characterized by high accumulation throughout the core followed by a drastic decrease of sedimentation near the top (Supplementary Fig 7a) as observed for PC23. In the inner-shelf of the Laptev Sea this drastic decrease in accumulation can be dated around 7,000 cal yBP which marks the end of the high sediment input. With increasing distance from the coast, this change in sedimentation progressively gets older because, in first-order approximation, the sediment deposition over the shelf essentially mirrored the sea level ingression and the migration of the sediment source. However, the timing of accumulation slightly differs between adjacent stations suggesting the presence of additional local accumulation patterns. In addition, differences can also be due to the different reservoir age and assumptions used to develop the age model.

In order to estimate the OC buried during the last phase of the deglaciation and early Holocene, as first step we constrained the volume of the deposit specifically focusing on the high accumulation transgressive period. The thickness of the transgressive deposit was defined as the interval between ca. 7,000 cal yBP and the bottom core (Supplementary Fig. 7a). In the attempt to make the upper boundary as consistent as possible among records, we selected the closest radiocarbon measurement to 7,000 cal yBP (yet older) for each core. Despite the fact that a few radiocarbon dates are older than 12,000 cal yBP, the deposit is mostly resolved between 7,000 and 14,000 cal yBP as shown by the histogram of the radiocarbon dates (Supplementary Fig. 7b). Finally, it is important to highlight that the differences in thickness between stations which are relatively close are likely due to the sediment

sampling technique – essentially driven by gravity - which can result in the incomplete recovery of the transgressive unit. Thus, the thickness of the deposit is likely underestimated.

The data interpolation has been performed using two different models in Matlab. For the first quantification (interpolation model 1, Supplementary Fig. 9a), all transgressive thicknesses were interpolated using a triangulation-based cubic method with a meshgrid of 10×10 km which extended for 20,450 km^2. In the second model (interpolation model 2, Supplementary Fig. 9b) we imposed (as a boundary conditions) that (i) the high flux period ended 7,000 years ago and (ii) the sediment accumulates at depths greater than 5 m which, in first-order approximation, corresponds to the modern -13m isobath (taking into account the average late-Holocene sediment thickness[3] as well as to the overall eustatic variation[27]). Using the latest bathymetry chart (IBCAO)[28] we extracted the 13 m isobath and imposed "no accumulation" along this line (Supplementary Fig. 10). For the second model, the 10×10 km meshgrid extended for 31,120 km^2. In order to limit our extrapolation within areas covered by the raw data, the meshgrid did not include the inner regions of the Laptev Sea (Bour-Khaya and Yanskij bays).

Once the volume of the deposit by each interpolation model was obtained, the total carbon buried was calculated by correcting for the average dry bulk density (1.21±0.19 g cm^{-3}) and the OC content (1.7±0.52 wt%). The dataset for the dry bulk density is composed of 343 data points (PC23 and other three sediment cores[4,6], PM9462-4, KD9502-14, PS2725-5; www.pangaea.de) while the bulk OC content was estimated using 381 measurements (PC23 and other five sediment cores[4,6], KD0502-14, PM9499-2, PS2458-4, PS2725-5; www.pangaea.de). Despite the different boundary conditions, both models returned comparable results: 16.4 and 16.8 Pg C for interpolation method 1 and 2, respectively. These estimates were averaged and the error was propagated using the uncertainties of the dry bulk density and bulk OC giving a final estimate of 16.6±5.7 Pg C.

Finally, by correcting for degradation after deposition and using the source apportionment results, we have estimated the Peta g of active-layer carbon originally supplied by rivers discharging into the

Laptev Sea during transgression. The loss of carbon due to degradation was considered to equal the difference between the modern average OC content in riverine suspended sediments (% $OC_{river\text{-}borne}$) and the average active-layer OC content remained in the transgressive unit after its deposition for several thousand years. The active-layer OC supplied to the Laptev Sea during transgression (31±9 Pg C) was therefore calculated using Eq.5

$$\text{Active layer OC supply} = \left(\text{buried OC}_{\text{bulk}} \times F_{\text{active layer}}\right) \times \left(\frac{\%OC_{\text{river-bome}}}{F_{\text{active layer}} \times \%OC_{\text{trangressive unit}}} \right) \qquad \text{(Eq.5)}$$

where buried OC_{bulk} is the cumulative bulk OC buried in the transgressive deposit previously calculated (16.6 Pg C), $F_{active\ layer}$ is the average fraction of active-layer permafrost in the transgressive deposit based on source apportionment calculations (ca. 0.75, Fig. 3), $\%OC_{transgressive\ unit}$ is the OC content left in the transgressive deposit which was previously used to assess the cumulative bulk OC buried (1.7%) and $\%OC_{river\text{-}borne}$ (3.2±0.8%) is the modern average OC content of Lena river suspended sediments[2,7,8] corresponding to Total Suspended Material (TSM) >30 mg l^{-1} as shown in Supplementary Fig. 11a. TMS values above >30 mg l^{-1} should reflect the actual sediment supplied during high flow (> 40,000 m^3 s^{-1}). By contrast, low-moderate discharge periods might be affected by in-situ production as suggested by the high OC content (up to 30%, Fig.11a). Because these low discharge periods are not representative in terms of carbon fluxes (Supplementary Fig.11c), TMS values below 30 mg l^{-1} were not included because they could have biased the degradation extent towards greater values.

Comparison with the modern river input

Over 70% of the sediment that accumulates in the Laptev Sea enters via the Lena river (20.7 Tg y^{-1}) while the rest is supplied by minor, local rivers (7.9 Tg y^{-1}; mainly from the Yana and Khatanga Rivers)[29]. The modern annual particulate OC (POC) flux by the Lena has been estimated between 0.38 and 1.2 Tg C[8,30]. The large difference between these figures likely lies on the different method used; the former estimate[30] (annual water discharge × POC) is based on a representative POC concentration (mg l^{-1}) measured during the freshet while the latter estimate[8] (annual sediment discharge × %OC)

relies on the average OC content (g dw,%) of the several TSM measurements in low and high discharge conditions.

As the river discharge at high latitudes exerts first-order control on TSM, POC and %OC, in this study we have developed a more elaborate system which models the carbon fluxes as a function of the discharge on the basis of the concept of rating curves[31]. This is essentially an empirical model which is commonly used to estimate the annual export of solid and dissolved constituents in rivers[32,33]. The rating curve for the Lena was built using the dataset provided by the Arctic Great Rivers Observatory[7] (Arctic-GRO, surface and depth-integrated measurements, Supplementary Fig. 11b) using Eq.6

$$POC = a \times Q^b \qquad \text{(Eq.6)}$$

where a and b are the regression coefficients[31] of the log-log plot POC (mgC l^{-1}) *vs* discharge (Q, $m^3\,s^{-1}$).

By applying the rating curve over the last 14 years of discharge data (Arctic-RIMS, http://rims/unh.edu) we assessed the daily and annual OC fluxes of the Lena River. According to our estimates, the average annual OC flux over this 14-y period is 0.49±0.12 Tg C y^{-1}. This suggests that the OC export reported in ref.7 which is based on the average OC content of suspended materials is likely overestimated. This is because low discharge periods (low TSM, Supplementary Fig 10a), despite the high %OC, supply much less OC than high discharge periods due to the considerably lower TSM and discharge (Supplementary Fig 11c).

The annual C flux calculated with the rating curve was then adjusted to account for the input by other minor Siberian rivers based on the annual sediment discharge to the Laptev Sea[29] using Eq.7

$$\text{annual OC flux to the Laptev Sea} = \text{annual OC flux}_{\text{LENA}} \times \left(\frac{\text{sediment input}_{\text{ALL RIVERS}}}{\text{sediment input}_{\text{LENA}}} \right) \qquad \text{(Eq.7)}$$

to yield a modern annual OC flux of 0.66 Tg C y^{-1}.

In order to compare the carbon supply in transgressive *vs* modern conditions, we have divided the active-layer OC supplied during transgression previously calculated (31±9 Pg C, Eq. 5) by the time interval during which the deposit was formed (where the deposit is better resolved from 7,000 to

14000 yBP, Supplementary Fig. 7). By doing so we obtained the average annual supply from the watershed adjacent to the Laptev Sea during transgression (4.5±1.4 Pg C y^{-1}). Assuming that the all modern river C input (Eq. 7) exclusively originates from the active-layer, we have estimated that the relative active-layer contribution during the late deglaciation/early Holocene period was 7 times higher than the modern input. It is important to highlight that this a conservative estimate because river-bank erosion of ICD occurs in the watershed[34] (which would therefore lower the modern active-layer contribution via river input) and our assessment did not include regions of the Laptev Sea where data are not available (Bour-Khaya and Yanskij bays, Supplementary Fig. 9 and 10).

SUPPLEMENTARY REFERENCES

1 Tesi, T., Semiletov, I., Hugelius, G., Dudarev, O., Kuhry, P. & Gustafsson, Ö. Composition and fate of terrigenous organic matter along the Arctic land–ocean continuum in East Siberia: Insights from biomarkers and carbon isotopes. *Geochimica et Cosmochimica Acta* **133**, 235-256 (2014).

2 Winterfeld, M., Goñi, M., Just, J., Hefter, J. & Mollenhauer, G. Characterization of particulate organic matter in the Lena River delta and adjacent nearshore zone, NE Siberia–Part 2: Lignin-derived phenol compositions. *Biogeosciences* **12**, 2261-2283 (2015).

3 Bauch, H. A., Mueller-Lupp, T., Taldenkova, E., Spielhagen, R. F., Kassens, H., Grootes, P. M., Thiede, J., Heinemeier, J. & Petryashov, V. Chronology of the Holocene transgression at the North Siberian margin. *Global and Planetary Change* **31**, 125-139 (2001).

4 Fahl, K. & Stein, R. Biomarkers as organic-carbon-source and environmental indicators in the Late Quaternary Arctic Ocean: problems and perspectives. *Marine Chemistry* **63**, 293-309 (1999).

5 Taldenkova, E., Bauch, H. A., Stepanova, A., Dem'yankov, S. & Ovsepyan, A. Last postglacial environmental evolution of the Laptev Sea shelf as reflected in molluscan, ostracodal, and foraminiferal faunas. *Global and Planetary Change* **48**, 223-251 (2005).

6 Mueller-Lupp, T., Bauch, H. A., Erlenkeuser, H., Hefter, J., Kassens, H. & Thiede, J. Changes in the deposition of terrestrial organic matter on the Laptev Sea shelf during the Holocene: evidence from stable carbon isotopes. *International Journal of Earth Sciences* **89**, 563-568 (2000).

7 http://www.arcticgreatrivers.org/data.html, A. G. R. O. A.-G.

8 Rachold, V. & Hubberten, H.-W. in *Land-Ocean Systems in the Siberian Arctic* 223-238 (Springer, 1999).

9 Andersson, A., Deng, J., Du, K., Zheng, M., Yan, C., Sköld, M. & Gustafsson, Ö. Regionally-Varying Combustion Sources of the January 2013 Severe Haze Events over Eastern China. *Environmental Science & Technology* **49**, 2038-2043, doi:10.1021/es503855e (2015).

10 Bosch, C., Andersson, A., Kruså, M., Bandh, C., Hovorková, I., Klánová, J., Knowles, T. D., Pancost, R. D., Evershed, R. P. & Gustafsson, O. r. Source Apportionment of Polycyclic Aromatic Hydrocarbons in Central European Soils with Compound-Specific Triple Isotopes (δ13C, Δ14C, and δ2H). *Environmental science & technology* **49**, 7657-7665 (2015).

11 Vonk, J., Sánchez-García, L., van Dongen, B., Alling, V., Kosmach, D., Charkin, A., Semiletov, I., Dudarev, O., Shakhova, N. & Roos, P. Activation of old carbon by erosion of coastal and subsea permafrost in Arctic Siberia. *Nature* **489**, 137-140 (2012).

12 Schirrmeister, L., Kunitsky, V., Grosse, G., Wetterich, S., Meyer, H., Schwamborn, G., Babiy, O., Derevyagin, A. & Siegert, C. Sedimentary characteristics and origin of the Late Pleistocene Ice Complex on north-east Siberian Arctic coastal lowlands and islands–a review. *Quaternary international* **241**, 3-25 (2011).

13 Jasinski, J., Warner, B., Andreev, A., Aravena, R., Gilbert, S., Zeeb, B., Smol, J. & Velichko, A. Holocene environmental history of a peatland in the Lena River valley, Siberia. *Canadian Journal of Earth Sciences* **35**, 637-648 (1998).

14 Kaiser, C., Meyer, H., Biasi, C., Rusalimova, O., Barsukov, P. & Richter, A. Conservation of soil organic matter through cryoturbation in arctic soils in Siberia. *Journal of Geophysical Research: Biogeosciences (2005–2012)* **112** (2007).

15 Höfle, S., Rethemeyer, J., Mueller, C. & John, S. Organic matter composition and stabilization in a polygonal tundra soil of the Lena Delta. *Biogeosciences* **10**, 3145-3158 (2013).

16 Palmtag, J., Hugelius, G., Lashchinskiy, N., Tamstorf, M. P., Richter, A., Elberling, B. & Kuhry, P. Storage, landscape distribution, and burial history of soil organic matter in contrasting areas of continuous permafrost. *Arctic, Antarctic, and Alpine Research* **47**, 71-88 (2015).

17 Andersen, K. K., Azuma, N., Barnola, J.-M., Bigler, M., Biscaye, P., Caillon, N., Chappellaz, J., Clausen, H. B., Dahl-Jensen, D. & Fischer, H. High-resolution record of Northern Hemisphere climate extending into the last interglacial period. *Nature* **431**, 147-151 (2004).

18 Gundelwein, A., Müller-Lupp, T., Sommerkorn, M., Haupt, E. T., Pfeiffer, E. M. & Wiechmann, H. Carbon in tundra soils in the Lake Labaz region of arctic Siberia. *European Journal of Soil Science* **58**, 1164-1174 (2007).

19 Bird, M., Santruckova, H., Arneth, A., Grigoriev, S., Gleixner, G., Kalaschnikov, Y., Lloyd, J. & Schulze, E. D. Soil carbon inventories and carbon-13 on a latitude transect in Siberia. *Tellus B* **54**, 631-641 (2002).

20 Panova, E., Tesi, T., Pearce, C., Salvado, J. A., Karlsson, E., Krusa, M., Semiletov, I. & Gustafsson, Ö. in *AGU Fall Meeting Abstracts.*

21 Hobson, K., Ambrose Jr, W. & Renaud, P. Sources of primary production, benthic-pelagic coupling, and trophic relationships within the Northeast Water Polynya: Insights from delta super (13) C and delta super (15) N analysis. *Marine ecology progress series. Oldendorf* **128**, 1-10 (1995).

22 Søreide, J. E., Hop, H., Carroll, M. L., Falk-Petersen, S. & Hegseth, E. N. Seasonal food web structures and sympagic–pelagic coupling in the European Arctic revealed by stable isotopes and a two-source food web model. *Progress in Oceanography* **71**, 59-87 (2006).

23 Schubert, C. J. & Calvert, S. E. Nitrogen and carbon isotopic composition of marine and terrestrial organic matter in Arctic Ocean sediments:: implications for nutrient utilization and organic matter composition. *Deep Sea Research Part I: Oceanographic Research Papers* **48**, 789-810 (2001).

24 Leu, E., Wiktor, J., Søreide, J., Berge, J. & Falk-Petersen, S. Increased irradiance reduces food quality of sea ice algae. *Marine Ecology Progress Series* **411**, 49-60 (2010).

25 Hobson, K. A. & Welch, H. E. Observations of foraging northern fulmars (Fulmarus glacialis) in the Canadian High Arctic. *Arctic*, 150-153 (1992).

26 Hobson, K. A., Fisk, A., Karnovsky, N., Holst, M., Gagnon, J.-M. & Fortier, M. A stable isotope (δ 13 C, δ 15 N) model for the North Water food web: implications for evaluating trophodynamics and the flow of energy and contaminants. *Deep Sea Research Part II: Topical Studies in Oceanography* **49**, 5131-5150 (2002).

27 Lambeck, K., Rouby, H., Purcell, A., Sun, Y. & Sambridge, M. Sea level and global ice volumes from the Last Glacial Maximum to the Holocene. *Proceedings of the National Academy of Sciences* **111**, 15296-15303 (2014).

28 Jakobsson, M., Mayer, L., Coakley, B., Dowdeswell, J. A., Forbes, S., Fridman, B., Hodnesdal, H., Noormets, R., Pedersen, R. & Rebesco, M. The international bathymetric chart of the Arctic Ocean (IBCAO) version 3.0. *Geophysical Research Letters* **39** (2012).

29 Gordeev, V. Fluvial sediment flux to the Arctic Ocean. *Geomorphology* **80**, 94-104 (2006).

30 Semiletov, I., Pipko, I., Shakhova, N., Dudarev, O., Pugach, S., Charkin, A., McRoy, C., Kosmach, D. & Gustafsson, Ö. Carbon transport by the Lena River from its headwaters to the Arctic Ocean, with emphasis on fluvial input of terrestrial particulate organic carbon vs. carbon transport by coastal erosion. *Biogeosciences* **8**, 2407-2426 (2011).

31 Asselman, N. Fitting and interpretation of sediment rating curves. *Journal of Hydrology* **234**, 228-248 (2000).

32 Hatten, J. A., Goñi, M. A. & Wheatcroft, R. A. Chemical characteristics of particulate organic matter from a small, mountainous river system in the Oregon Coast Range, USA. *Biogeochemistry* **107**, 43-66 (2012).

33 Wheatcroft, R. A., Hatten, J. A., Pasternack, G. B. & Warrick, J. A. The role of effective discharge in the ocean delivery of particulate organic carbon by small, mountainous river systems. *Limnology and Oceanography* **55**, 161-171 (2010).

34 Winterfeld, M., Laepple, T. & Mollenhauer, G. Characterization of particulate organic matter in the Lena River delta and adjacent nearshore zone, NE Siberia – Part I: Radiocarbon inventories. *Biogeosciences* **12**, 3769-3788, doi:10.5194/bg-12-3769-2015 (2015).
